# RAI3 expression is not associated with clinical outcomes of patients with non-small cell lung cancer

**DOI:** 10.1007/s00432-023-04631-3

**Published:** 2023-02-13

**Authors:** Nathaniel Melling, Matthias Reeh, Tarik Ghadban, Michael Tachezy, André Hajek, Jakob Robert Izbicki, Katharina Grupp

**Affiliations:** 1 General, Visceral and Thoracic Surgery Department and Clinic, University Medical Center Hamburg-Eppendorf, Hamburg, Germany; 2 Department of Health Economics and Health Services Research, University Medical Center Hamburg-Eppendorf, Hamburg, Germany; 3 Department of Plastic, Reconstructive and Aesthetic Surgery, University Medical Center Hamburg-Eppendorf, Hamburg, Germany

**Keywords:** RAI3, Lung adenocarcinoma, Squamous cell lung carcinoma, Large cell lung carcinoma, Immunohistochemistry, Non-small cell lung cancer

## Abstract

**Purpose:**

Retinoic acid inducible protein 3 (RAI3) has been suggested as prognostic biomarker in several cancer types. The present study aimed to examine the role of RAI3 expression in non-small cell lung cancers (NSCLCs).

**Methods:**

RAI3 protein expression was evaluated by immunohistochemistry in tissue microarray (TMA) sections from a retrospective cohort of more than 600 surgically resected NSCLCs and results were compared with clinicopathological features and follow-up data.

**Results:**

While membranous RAI3 immunostaining was always strong in benign lung, strong RAI3 staining was only detectable in 14.7% of 530 interpretable NSCLCs. Within NSCLC subtypes, immunostaining intensity for RAI3 was significantly decreased in large cell lung cancers (LCLCs) and squamous cell carcinomas (SQCCs) relative to lung adenocarcinomas (LUACs) (*P* < 0.0001 each). However, RAI3 staining was neither associated with pathological features of NSCLCs nor with survival of patients (*P* = 0.6915).

**Conclusion:**

Our study shows that RAI3 expression was not associated with clinical outcomes of NSCLC patients and cannot be considered as prognostic marker in lung cancer patients.

## Introduction

Non-small cell lung cancer (NSCLC) is a heterogeneity disease and to date, specific clinical factors and tumor stage are established as prognostic markers. Nevertheless, prognosis within stage may vary significantly. Therefore, the identification of novel biomarkers reflecting the early diagnosis and exact assessment of individual patient prognosis and thus enable better decision-making in the individual treatment of NSCLC patients are desperately needed.

The retinoic-acid inducible protein 3 (RAI3), also known as G-protein-coupled receptor family C group 5 type A (GPRC5A) consists of extracellular ligand-binding-, transmembrane, and internal C-terminal domain (Li et al. 2005). The intracellular C-terminus interacts with G-proteins that bind guanidine-nucleotides and can activate downstream effectors such as adenyl cyclases, phospholipases, phosphodiesterases and ion channels when an agonist binds to the extracellular portion of the receptor (McCudden et al. 2005). GPCRs activate numerous signal transduction cascades and thus play pivotal role in the regulation of many physiological processes, including cell growth and differentiation (Shore and Reggio 2015).

Previous studies have suggested that dysregulation of RAI3 expression is associated with several malignancies, including increased RAI3 expression in colorectal (Kume et al. 2014; Zougman et al. 2013) and breast cancer (Jörißen et al. 2009) or decreased RAI3 expression in oral squamous cell (Liu et al. 2013), non-small cell lung (Fujimoto et al. 2012), hepatocellular (Zheng et al. 2014), and gastric cancer (Cheng et al. 2012).

For lung, RAI3 overexpression resulted in reduced cell growth in NSCLC cell line H1792 (Xu et al. 2005), *GPRCA5*-knockout mice developed spontaneous lung tumors (Tao et al. 2007) and loss of heterozygosity of chromosome 12p, where GPRC5A gene resides has been frequently detected in human NSCLCs (Takeuchi et al. 1996; Grepmeier et al. 2005). In addition, reduced RAI3 protein expression has been suggested in malignant relative to benign lung (Fujimoto et al. 2012; Lin et al. 2014). To further examine the role of RAI3 immunostaining in NSCLCs, we took advantage of our TMA containing of more than 600 lung cancer specimens with follow-up data. Our study shows that RAI3 expression was not associated with prognosis of NSCLC patients.

## Patients and methods

### Patient cohort and TMA construction

Lung cancer TMAs with a total of 619 NSCLC specimens were included in this study. Patients were treated at the University Medical Center Hamburg-Eppendorf, Germany. The TMAs contained 619 NSCLC specimens, including 227 LUACs, 134 LCLCs, and 258 lung SQCCs. Raw survival data were obtained from the responsible physicians for 300 patients. The median follow-up time was 18.3 months (range 0–155 months). TMA construction was as described (Mirlacher and Simon 2010). In brief, hematoxylin and eosin-stained sections were made from each block to define representative tumor regions. One tissue cylinder with a diameter of 0.6 mm was then punched from the tumor on the “donor” tissue block using a home-made semi-automated precision instrument and brought into empty recipient paraffin blocks. Four µm sections of the resulting TMA blocks were transferred to an adhesive coated slide system (Instrumedics Inc., Hackensack, New Jersey). The usage of tissue microarrays for research purposes has been approved by the local ethics committee.

### Immunohistochemical staining

Freshly cut TMA sections were analyzed on 1 day and in one experiment. Slides were deparaffinized and exposed to heat-induced antigen retrieval for 5 min in an autoclave at 121 C in pH 7.8 Tris–EDTA-Citrate buffer. Primary antibody specific for RAI3 (polyclonal rabbit, NB100-310; Novus Biological; at 1/450 dilution) was applied at 37 °C for 60 min. Bound antibody was then visualized using the EnVision Kit (Dako, Glostrup, Denmark) according to the manufacturer’s directions. RAI3 staining was analyzed by one person (KG) experienced in immunohistochemisty. Assessment of immunostaining was performed in four categories: negative, weak, moderate, and strong immunostaining.

### Statistical analysis

Statistical calculations were performed with JMP^®^ 10.0.2 software (2012 SAS Institute Inc., NC, USA). Contingency tables and the chi-square test were performed to search for associations between molecular parameters and tumor phenotype. Survival curves were calculated according to Kaplan–Meier. The Log-Rank test was applied to detect significant survival differences between groups. Cox proportional hazards regression analysis was performed to test the statistical independence and significance between pathological, molecular and clinical variables.

## Results

### Prevalence of RAI3 immunostaining in NSCLCs

**Fig. 1:**
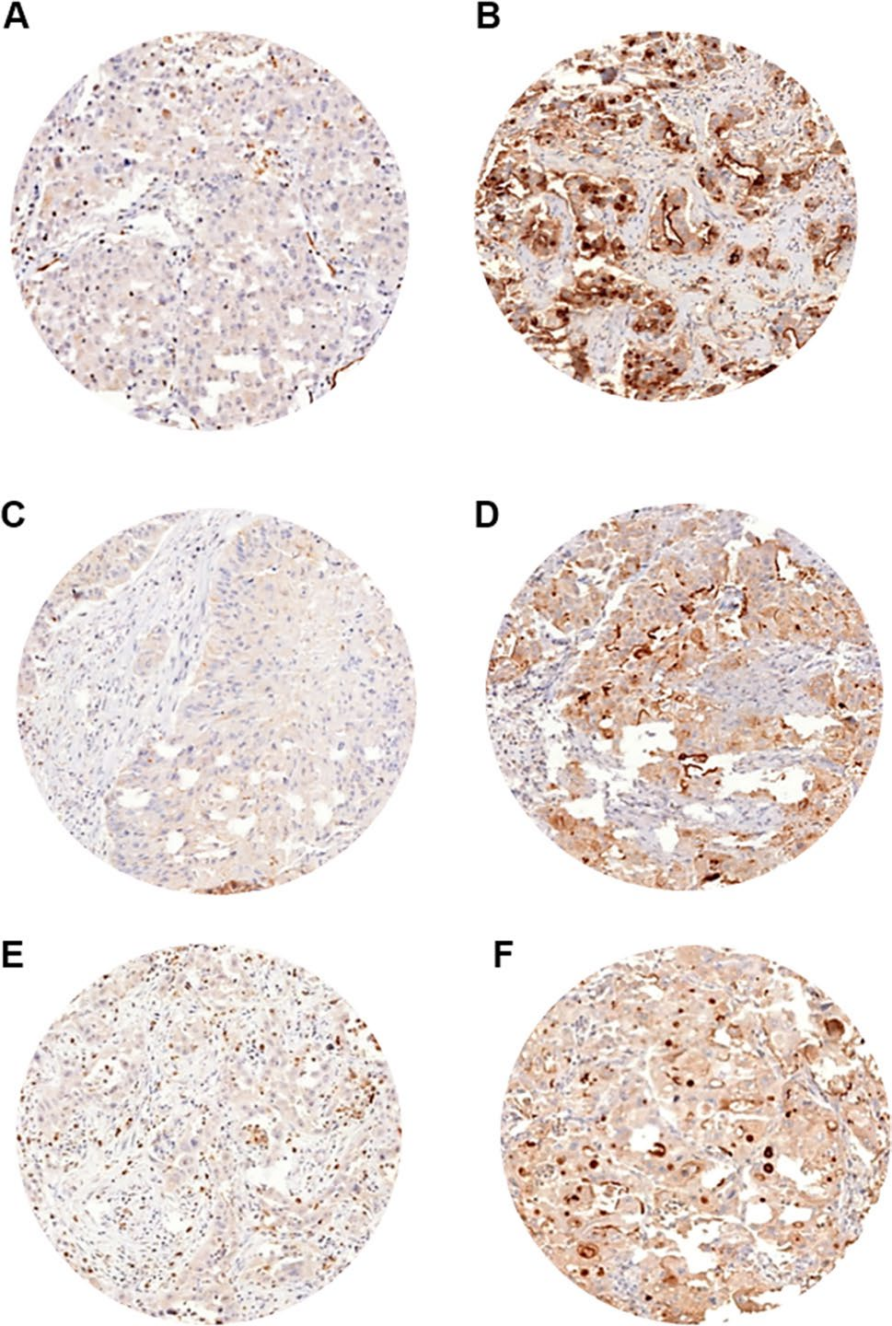
RAI3 immunohistochemical membranous protein expression in NSCLC subtypes. Immunohistochemical examples of negative and strong RAI3 immunostaining in LUACs, LCLCs, and SQCCs

**Fig. 2:**
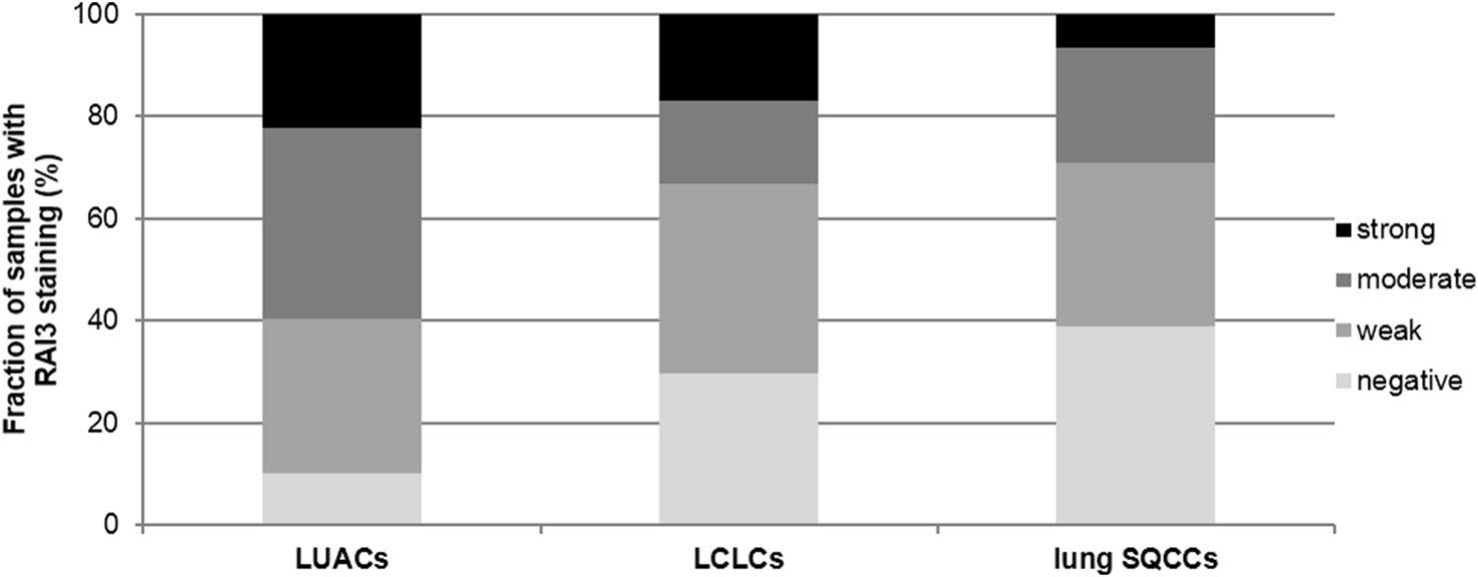
Frequency of RAI staining intensities in NSCLC subtypes. Immunostaining intensity for RAI3 was decreased in LCLCs and SQCCs relative to LUACs (*P* < 0.0001 each)

We obtained interpretable staining results for RAI3 in a total of 530 of 619 (84.1%) tumor samples. The reason for failure was insufficient tumor cells available for scoring. Immunoreactivity for RAI3 was predominantly detectable in the membranes of the cells. Immunostaining intensity for RAI3 was always strong in benign lung and was decreased in tumors relative to normal tissue. In NSCLCs, positive RAI3 staining was only observed in 73.6% of 530 interpretable NSCLCs, including 32.3% with weak, 26.6% with moderate, and 14.7% with strong staining. Representative images of RAI3 immunohistochemical membranous protein expression are given in Fig. 1. Within NSCLCs, RAI3 expression was significantly lower in LCLCs and SQCCs than in LUACs (*P* < 0.0001 each) as visualized in Fig. 2.

### Prognostic significance of RAI3 immunostaining in NSCLCs

In the analysis of all NSCLCs, RAI3 staining was not strongly linked to pathological tumor stage (*P* = 0.3012), lymph node and distant metastasis status (*P* = 0.1398 and *P* = 0.0816) as shown in Table 1. Additionally, there were no significances observed between RAI3 staining and clinicopathological features in histological subsets of NSCSLs (Table 2).

**Table 1:** Correlation between RAI3 immunostaining and clinicopathological features of NSCLCs

Clinicopathologic parameter	*n* on TMA	RAI3 immunostaining					*P* value
		*n* evaluable	(%) negative	(%) weak	(%) moderate	(%) strong	
All cancers	619	530	26.42	32.26	26.6	14.72	
Histology							
Adenocarcinoma	227	196	10.2	30.1	37.24	22.45	< 0.0001
Large cell carcinoma	134	111	29.73	36.94	16.22	17.12	
Squamous cell carcinoma	258	223	39.01	31.84	22.42	6.73	
pT stage							
pT1	174	140	19.29	33.57	32.14	15	0.3021
pT2	323	291	28.52	31.62	25.43	14.43	
pT3	53	46	30.43	30.43	21.74	17.39	
pT4	50	43	34.88	37.21	13.95	13.95	
N status							
pN0	264	227	22.91	32.6	28.63	15.86	0.1398
pN1	164	146	34.25	31.51	23.29	10.96	
pN2	111	94	27.66	38.3	20.21	13.83	
pN3	18	16	25	43.75	6.25	25	
M status							
M0	577	500	27.4	32.4	25.8	14.4	0.0816
M1	33	25	8	32	36	24

**Table 2:** Correlation between RAI3 immunostaining and clinicopathological features of (A) LUACs, (B) LCLCs, and (C) SQCCs

(A)						
Clinicopathologic parameter	RAI3 immunostaining					*P* value
	*n* evaluable	(%) Negative	(%) Weak	(%) Moderate	(%) Strong	
Tumors	196	10.2	30.1	37.24	22.45	
pT stage						
pT1	67	7.46	32.84	46.27	13.43	0.4229
pT2	95	11.58	30.53	29.47	28.42	
pT3	13	7.69	30.77	30.77	30.77	
pT4	13	15.38	23.08	38.46	23.08	
N status						
pN0	94	10.64	31.91	35.11	22.34	0.6772
pN1	36	8.33	33.33	38.89	19.44	
pN2	34	11.76	38.24	23.53	26.47	
pN3	5	20	40	0	40	
M status						
M0	178	10.67	31.46	36.52	21.35	0.0901
M1	13	0	15.38	38.46	46.15	

(B)						
Clinicopathologic parameter	RAI3 immunostaining					*P* value
	*n* evaluable	(%) Negative	(%) Weak	(%) Moderate	(%) Strong	
Tumors	111	29.73	36.94	16.22	17.12	
pT stage						
pT1	33	21.21	36.36	15.15	27.27	0.5218
pT2	58	34.48	39.66	17.24	8.62	
pT3	9	22.22	33.33	22.22	22.22	
pT4	11	36.36	27.27	9.09	27.27	
N status						
pN0	51	27.45	35.29	19.61	17.65	0.97
pN1	23	26.09	47.83	8.7	17.39	
pN2	19	21.05	42.11	15.79	21.05	
pN3	8	37.5	37.5	12.5	12.5	
M status						
M0	109	29.36	37.61	15.6	17.43	0.3417
M1	2	50	0	50	0	

C)						
Clinicopatholgic parameter	RAI3 immunostaining					*P* value
	*N* evaluable	(%) Negative	(%) Weak	(%) Moderate	(%) Strong	
Tumors	223	39.01	31.84	22.42	6.73	
pT stage						
pT1	40	37.5	32.5	22.5	7.5	0.0743
pT2	138	37.68	28.99	26.09	7.25	
pT3	24	45.83	29.17	16.67	8.33	
pT4	19	47.37	52.63	0	0	
N status						
pN0	82	34.15	31.71	26.83	7.32	0.0799
pN1	87	47.13	26.44	20.69	5.75	
pN2	41	43.9	36.59	19.51	0	
pN3	3	0	66.67	0	33.33	
M status						
M0	213	40.38	30.52	22.07	7.04	0.0755
M1	10	10	60	30	0	

**Fig. 3:**
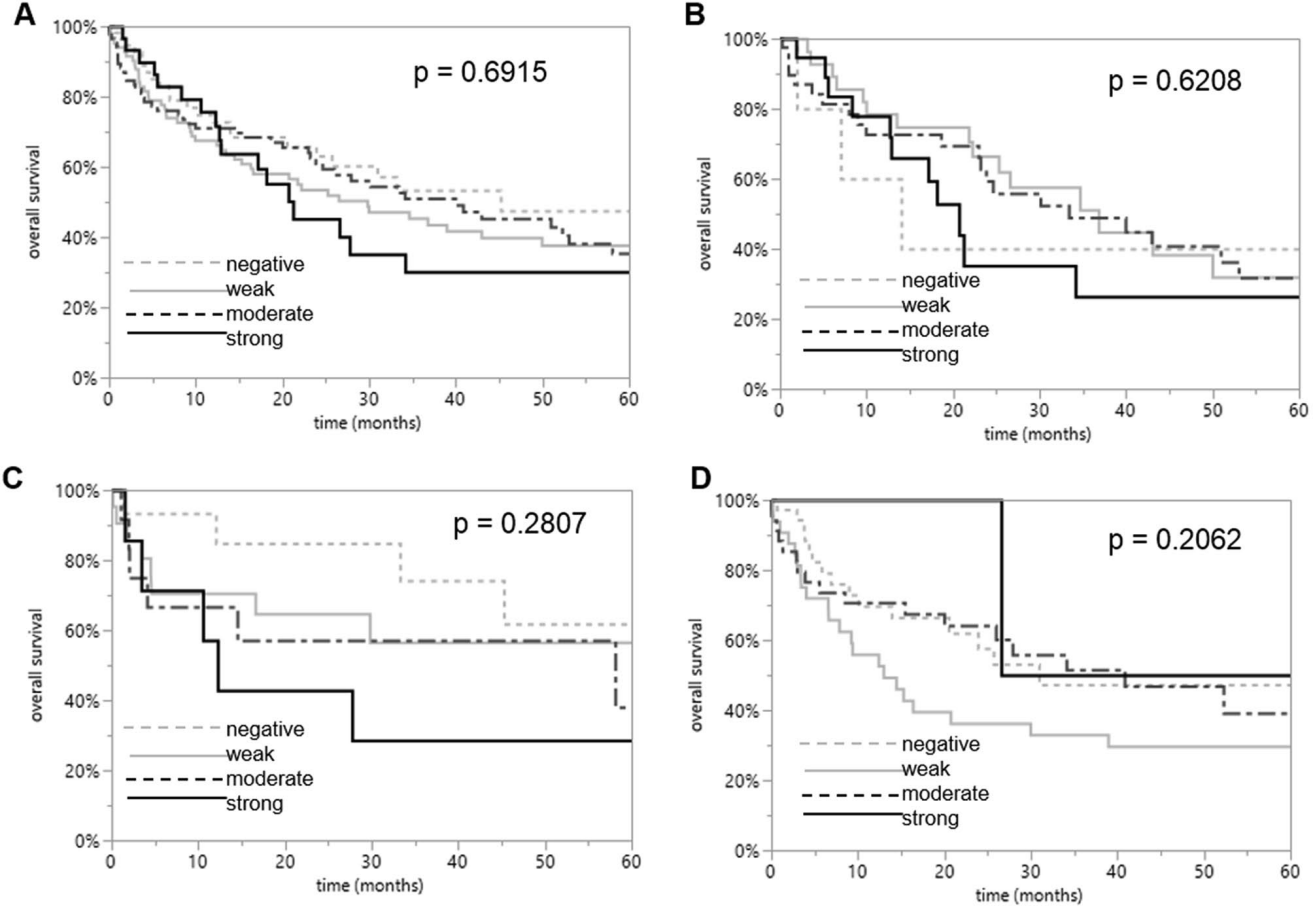
Clinical impact of RAI3 in NSCLCs. Kaplan–Meier plots for the overall survival rate in all NSCLC patients (*P* = 0.6915) and in subsets of LUAC (*P* = 0.6208), LCLC (*P* = 0.2807), and SQCC (*P* = 0.2062) patients

Survival curves demonstrated that RAI3 expression was unrelated to outcome if all NSCLCs (*P* = 0.6915) or subsets of LUACs (*P* = 0.6208), LCLCs (*P* = 0.2807), and SQCCs (*P* = 0. 0.2062) were analyzed (Fig. 3).

## Discussion

The successful analysis of RAI3 expression revealed that RAI3 expression was decreased in malignant as compared to benign tissue. A comparison with clinicopathological parameters of tumor aggressiveness did not suggest a clinical significance of RAI3 expression in lung cancers.

Here, we identify lower levels of RAI3 immunostaining in lung cancers than normal bronchial epithelia. This result is in line with previous studies analyzing RAI3 expression in cohorts of 150 and 474 NSCLC patients (Lin et al. 2014; Fujimoto et al. 2012). The functional relevance of RAI3 expression in benign lung was previously highlighted by earlier studies demonstrating that *GPRC5A-*knockout mice spontaneous develop lung adenomas and adenocarcinomas (Tao et al. 2007) and that loss of heterozygosity of chromosome 12p13 is a common alteration in NSCLCs (Takeuchi et al. 1996; Grepmeier et al. 2005). Taken together, it is tempting to suggest that GPRC5A might have a tumor-suppressive function in lung epithelial cells.

Within NSCLC subtypes, RAI3 expression was significantly decreased in LCLCs and SQCCs relative to LUACs. This observation is in accordance with the study of Fujimoto et al*.* (Fujimoto et al. 2012) on RAI3 expression in a cohort of 474 NSCLC patients, describing a positive association between RAI3 expression and LUAC histology (Fujimoto et al. 2012). Moreover, Fujimoto et al*.* (Fujimoto et al. 2012) suggested that RAI3 expression was highest in disease-free lung, decreased and intermediate in lung of cancer-free COPD patients and further attenuated and lowest in epithelia of COPD patients with LUAC and SQCC histology. These findings pinpoint to a potential tumor-suppressive role, similar to that established in mice, of *GPRC5A* in the sequential development of human NSCLC, in particular those associated with inflammatory chronic obstructive disease which is a major risk factor for lung cancer and shares various pathogenic features with lung tumor (Wistuba and Gazdar 2006).

The mechanism how RAI3 drives lung carcinogenesis remains elusive. Interestingly, *GPRC5A-*knockout mice developed LUACs and not LCLCs and SQCCs (Tao et al. 2007). These findings may be due to biological pathway-specific gene signatures that are differentially expressed and relevant in distinct subtypes of NSCLC. Previous microarray analysis showed that the transcriptomes of lung epithelial cells of *GPRC5A-*knockout defined a loss-of-*GPRC5A* gene signature, which was characterized by many aberrations in cancer-associated pathways, and was prevalent in human LUACs compared with SQCCs or normal lung (Wistuba and Gazdar 2006).

In literature, RAI3 has been suggested as prognostic marker in several cancer types, including colon cancer (Kume et al. 2014; Zougman et al. 2013), gastric cancer (Cheng et al. 2012), oral squamous cell carcinoma (Liu et al. 2013), and hepatocellular carcinoma (Zheng et al. 2014). In contrast, we were not able to find a prognostic significance of RAI3 expression.

In summary, these results exclude RAI3 as prognostic marker but underlines the potential role of RAI3 as tumor suppressor in lung cancer.

## Data Availability

The datasets generated during and/or analyzed during the current study are available from the corresponding author on reasonable request.

## Abbreviations

NSCLC: Non-small cell lung cancer
RAI3: Retinoic acid inducible protein 3
LCLCs: Large cell lung cancers
SQCCs: Squamous cell carcinomas
LUACs: Lung adenocarcinomas
GPRC5A: G-protein-coupled receptor family C group 5 type A
TMA: Tissue microarray
